# Preanalytical Conditions and DNA Isolation Methods Affect Telomere Length Quantification in Whole Blood

**DOI:** 10.1371/journal.pone.0143889

**Published:** 2015-12-04

**Authors:** Alexander Tolios, Daniel Teupser, Lesca M. Holdt

**Affiliations:** Institute of Laboratory Medicine, Ludwig-Maximilians-University Munich, Munich, Germany; University of Newcastle, UNITED KINGDOM

## Abstract

Telomeres are located at chromosome ends and their length (TL) has been associated with aging and human diseases such as cancer. Whole blood DNA is frequently used for TL measurements but the influence of preanalytical conditions and DNA isolation methods on TL quantification has not been thoroughly investigated. To evaluate potential preanalytical as well as methodological bias on TL, anonymized leftover EDTA-whole blood samples were pooled according to leukocyte counts and were incubated with and without actinomycin D to induce apoptosis as a prototype of sample degradation. DNA was isolated from fresh blood pools and after freezing at -80°C. Commercially available kits using beads (Invitrogen), spin columns (Qiagen, Macherey-Nagel and 5prime) or precipitation (Stratec/Invisorb) and a published isopropanol precipitation protocol (IPP) were used for DNA isolation. TL was assessed by qPCR, and normalized to the single copy reference gene *36B4* using two established single-plex and a new multiplex protocol. We show that the method of DNA isolation significantly affected TL (e.g. 1.86-fold longer TL when comparing IPP vs. Invitrogen). Sample degradation led to an average TL decrease of 22% when using all except for one DNA isolation method (5prime). Preanalytical storage conditions did not affect TL with exception of samples that were isolated with the 5prime kit, where a 27% increase in TL was observed after freezing. Finally, performance of the multiplex qPCR protocol was comparable to the single-plex assays, but showed superior time- and cost-effectiveness and required > 80% less DNA. Findings of the current study highlight the need for standardization of whole blood processing and DNA isolation in clinical study settings to avoid preanalytical bias of TL quantification and show that multiplex assays may improve TL/SCG measurements.

## Introduction

Telomeres are DNA sequences defining the ends of chromosomes [1]. They are present in almost all species with linear chromosomes [2] and consist of repetitive hexameres (TTAGGG)_n_ oriented from 5’ to 3’ as well as a heterogeneous group of associated telomere-binding proteins [3]. Their size is highly dynamic spanning from less than 500 bp to more than 20 kbp [4]. Telomere shortening has been suggested as an intrinsic clock [5], limiting somatic cell divisions (known as the “Hayflick limit” [6]) before entering the stage of “replicative senescence” [3, 7]. Decreased telomere length has also been associated with obesity and smoking [8, 9], as well as genomic instability [10]. Moreover, an association of decreased telomere length with several diseases, such as increased risk of cancer [11], idiopathic pulmonary fibrosis [12], bone marrow failure and/or liver cirrhosis [13], acute myeloid leukemia, and myelodysplastic syndrome [14–16] has been demonstrated.

For assessment of telomere length, different methods have been established. The first technique was the Terminal Restriction Fragment (TRF) length analysis by Southern blot gel electrophoresis [4, 17]. This method utilizes restriction enzymes to fully digest genomic DNA while sparing telomeres due to their repetitive sequences, resulting in short genomic DNA pieces and long telomeric sequences. Although this technique is still considered the gold standard for telomere analysis, it has several limitations and disadvantages, for example the high DNA amounts required for the analysis as well as the complex and time-consuming methodology [2]. Alternative approaches were aimed to overcome these limitations, such as the Single TElomere Length Analysis (STELA), a single molecule ligation PCR-based method [18], and quantitative fluorescence in situ hybridization (Q-FISH) using digital fluorescence microscopy [19–21].

To facilitate high-throughput telomere length measurements, PCR-based assays were developed which normalize TL to a single copy gene [2, 22]. Although this method is fast, scalable [20], and the cost per sample is significantly lower compared to TRF, standardization is difficult and results from different laboratories may not be directly compared [23, 24]. Furthermore, there is evidence that qPCR-based TL quantification may also be affected by DNA isolation methods [25].

Therefore, it was the aim of the current study to evaluate the effect of five different commercial DNA isolation kits (from Invitrogen, Qiagen, Macherey-Nagel, 5prime and Stratec/Invisorb) as well as one published isopropanol precipitation protocol (IPP) in combination with different preanalytical sample treatments, such as sample freezing and degradation, on telomere length. TL was analyzed with an established single-plex protocol, normalizing TL to the single copy reference gene *36B4* [22] and compared to a novel, multichrome multiplex assay allowing high throughput parallel analysis

## Materials and Methods

### Study design and sample preparation

**Fig 1:**
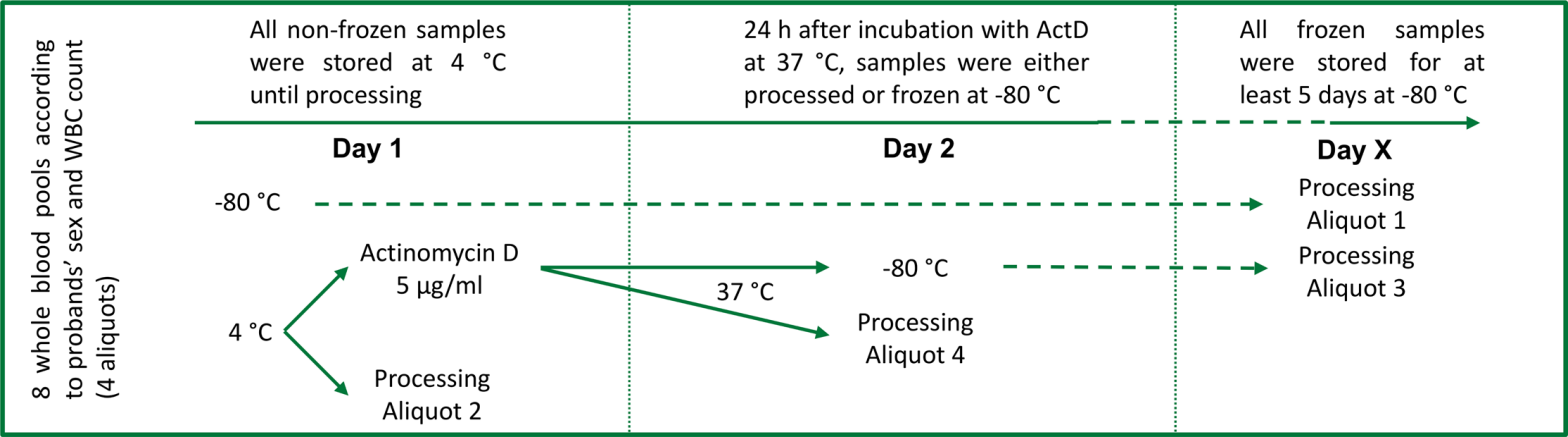
Study design. Leftover EDTA-whole blood samples were pooled according to the patients’ sex and leukocyte counts (n = 8). Each pool was divided in 4 aliquots (n = 32). The first aliquot (Aliquot 1; n = 8) was directly frozen. The second aliquot (Aliquot 2; n = 8) was processed directly without incubation (Day 1), while the rest was stimulated with actinomycin D (5 μg/ml). After 24 hours (Day 2), the third aliquot (Aliquot 3; n = 8) was frozen, while the last aliquot (Aliquot 4; n = 8) was processed. Before freezing, a white blood cell count was performed. Frozen aliquots (Aliquots 1 and 3) were processed within 5 to 14 days (Day X).

Anonymized left-over whole blood from routine laboratory analyses from 800 patients was pooled. Guidelines of the LMU ethics comittee do not require a specific ethics statement for this type of study. Samples were pooled at the day of collection according to patients’ sex and white blood cell numbers classified in quartiles according to absolute leukocyte counts (4 male pools and 4 female pools; Fig 1, S1 Fig). No significant differences were found with respect to age of patients composing the different pools (data not shown). A cell count was performed in all pools using a Sysmex XT 2000i automated hematology analyzer and pools were kept in movement at 4°C until processing or freezing. Each pool was divided in 4 aliquots, of which two were incubated with 5 μg/ml actinomycin D for 24 hours at 37°C to induce apoptosis [26, 27] as well as subsequent DNA degradation [28, 29] as previously described. After incubation, an additional cell count was performed (S1 Fig). Two aliquots per pool (one with and one without actinomycin D treatment) were processed directly, while the remaining two aliquots were stored at -80°C for 5 to 14 days before DNA isolation. Frozen samples were thawed for 30 minutes before DNA isolation. The study design is summarized in Fig 1.

### DNA isolation

**Fig 2:**
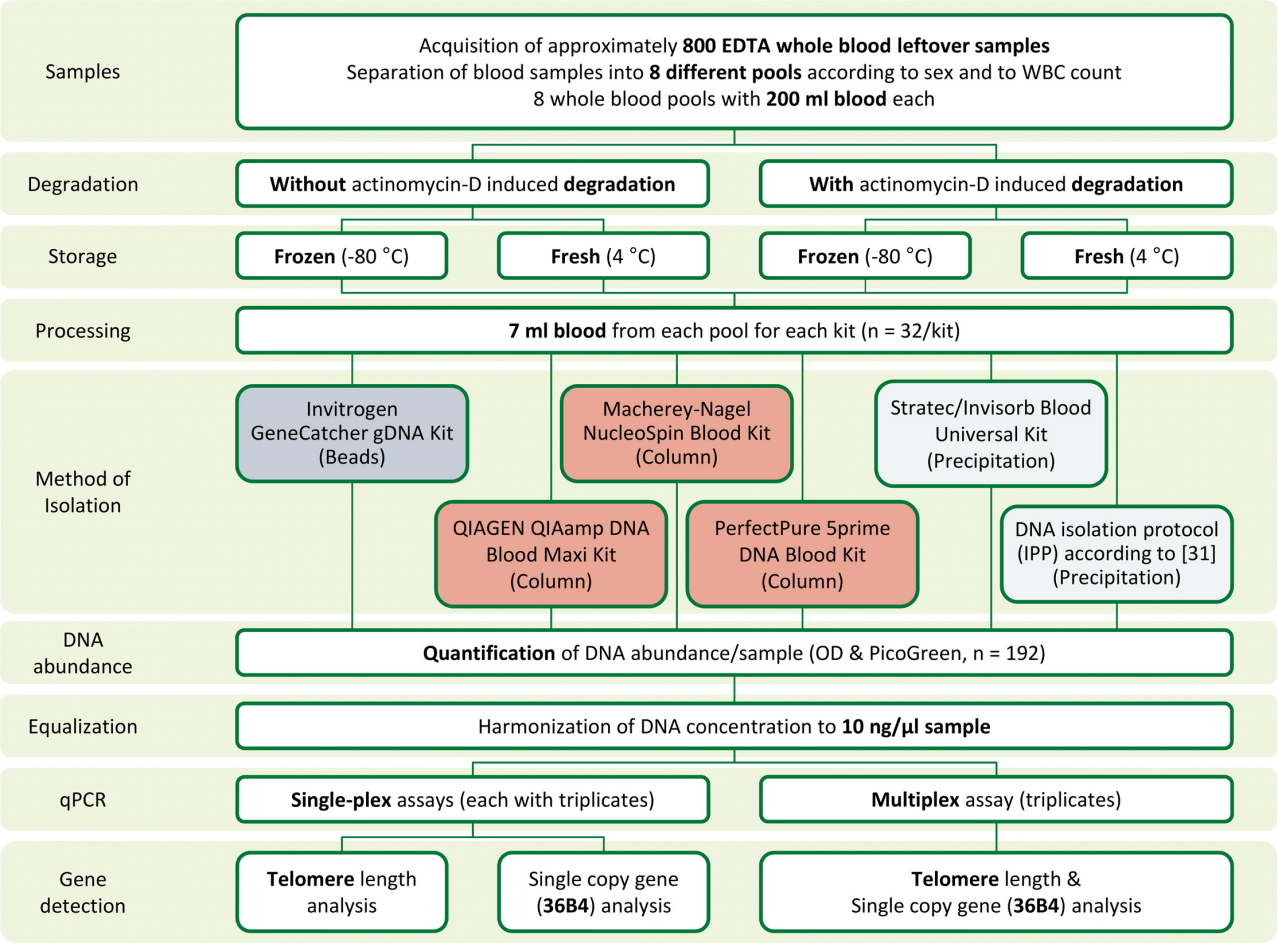
Sample preparation and analysis procedure. For comparison of preanalytical conditions and DNA isolation kits, EDTA-whole blood aliquots (8 pools, each in 4 different conditions) were processed using 6 different procedures. DNA concentration was harmonized in all 192 samples to 10 ng/μl and qPCR was performed with 1 μl sample in a total reaction volume of 10 μl. Each sample was analyzed using both a multiplex and a single-plex assay for telomere length quantification.

DNA isolation was performed using 7 ml aliquots of fresh or frozen whole blood (Fig 2; n = 192). Maxi DNA extraction kits from 5 different suppliers (GeneCatcher gDNA Kit from Invitrogen, QIAamp DNA Blood Maxi Kit from Qiagen, NucleoSpin Blood XL Kit from Macherey-Nagel, PerfectPure DNA Blood Kit from 5prime and Invisorb Blood Universal Kit from Stratec) as well as one published isopropanol precipitation protocol (IPP) [30, 31] were used. DNA extraction kits used magnetic beads (Invitrogen), large (Marcherey-Nagel, Qiagen) and small (5prime) spin columns or precipitation (Stratec, IPP). DNA isolation was performed according to the manufacturers’ instructions and DNA was eluted in 300 μl (5prime kit), 600 μl (IPP), 1000 μl (Marchery-Nagel, Qiagen), 1400 μl (Stratec) or 1500 μl (Invitrogen). After elution, DNA was quantified using OD measurements performed in duplicates for each sample on a NanoDrop ND-1000 spectrophotometer (Fisher Scientific) and was stored at -20°C until further use. An overview about the characteristics of the kits can be found in Table 1. Before qPCRs, samples were diluted to a DNA concentration of 10 ng/μl and quantified with the Quant-iT PicoGreen dsDNA Assay Kit (Invitrogen) on a SpectraMax Paradigm Multi-Mode Microplate Detection Platform (Molecular Devices) before subsequent analyses. One sample was lost due to handling errors.

**Table 1:** Specifications of DNA isolation methods and required laboratory equipment.

	Invitrogen GeneCatche gDNA Kit	Qiagen QIAamp DNA Blood Maxi Kit	Macherey-Nagel NucleoSpin Blood Kit	5prime PerfectPure DNA Blood Kit	Stratec/ Invisorb Blood Universal Kit	DNA isolation protocol (IPP) according to [31]
**Method**	Beads	Spin Column Large	Spin Column large	Spin Column small	Precipitation	Precipitation
**Hands-on time**	1.5 hours	1.5 hours	1.5 hours	1.5 hours	1.5 hours	2 hours
**Incubation/ elution times**	1 hour	-	-	-	1 hour	overnight incubation
**Yield according to supplier**	300 μg	15–600 μg	200–300 μg	150–250 μg	up to 400 μg	up to 500 μg
**Suggested elution volume**	1000–1500 μl	1000 μl	1000 μl	300 μl	1400 μl	300–600 μl
**Expected DNA fragment size**	larger than 50 kb	100 bp up to 50 kb, maximum at 30 kb	200 bp until approx. 50 kb	larger than 50 kb	no data available	no data available
**Centrifuge (50 ml tubes)**	-	x	x	x	x	x
**Centrifuge (1.5 ml tubes)**	-	-	-	x	-	-
**Water bath (50 ml tubes)**	x	x	x	-	-	x
**Thermomixer(50 ml tubes)**	-	-	-	-	x	x
**Thermomixe (1.5 ml tubes)**	-	-	X	-	-	-
**Additionally required equipment**	Magnetic Rack for 50 ml tubes	-	-	-	-	-

“x”- required; “-“—not required.

### Quantitative PCR

For TL measurement, a qPCR-based method was adapted from the protocol used by Cawthon [22]. Quantification was performed using the SensiMix SYBR No-ROX Kit (Bioline) and Ct-values were calculated according to Pfaffl [32]. Data were normalized to the single copy gene (SCG) *36B4*, which was quantified using published primers [22, 31] and fold changes were calculated accordingly. Characteristics of both assays are provided in Table 2. qPCRs were performed in 384-well plates on a ViiA7 (Life Technologies). A multiplex-assay was established for simultaneous measurement of TL and SCG in a single well. To this end, a probe with 5’-LC610 fluorophore and 3’ BHQ-2 labelling (Eurofins-MWG) was used to detect the single copy gene. The sequences for probe and primers are as following: Tel1b: 5’- CGG TTT GTT TGG GTT TGG GTT TGG GTT TGG GTT TGG GTT -3’; Tel2b: 5’- GGC TTG CCT TAC CCT TAC CCT TAC CCT TAC CCT TAC CCT -3’; 36B4 fwd: 5’- CAG CAA GTG GGA AGG TGT AAT CC -3’; 36B4 rev: 5’- CCC ATT CTA TCA TCA ACG GGT ACA A -3’; 36B4 probe: 5’- LC610—CGG ATT TCT TCA GCT TGT GCT TGT CTC CCT—BHQ2–3’. Due to the different light absorption and emission maxima from SYBR green I (495 nm and 520 nm, respectively) and LC610 (590 nm and 610 nm, respectively), telomeres and SCG were detected simultaneously without signal interferences when performing qPCR.

**Table 2:** Comparison of a published single-plex assays for quantification of TL and SCG and multiplex assay (adapted from [22, 31]).

	Single-plex assay according to [31]	Multiplex assay	Advantages of multiplex assay
**Telomere analysis**	25 μl/reaction with 12.5 μl Taq-Pol MM*	10 μl/reaction with 5 μl Taq-Pol MM*	80% reduction of Taq-Pol MM*
**Single Copy Gene analysis**	25 μl/reaction with 12.5 μl Taq-Pol MM*	LC610-labelled probe added to TL assay	Identical input DNA, thus better standardization
**Sample DNA required**	30 ng DNA/reaction (2 assays x triplicate values = 180 ng DNA)	10 ng DNA (multiplex assay in triplicates = 30 ng DNA)	> 80% less DNA required for parallel TL and SCG measurement
**Throughput**	32 samples on Rotorgene Q (Qiagen) in triplicates according to [31]	384well plate compatibility, 126 samples in triplicates	4x higher throughput
**Time on thermal cycler**	~ 80 minutes/reaction (2 reactions = 160 minutes)	~ 50 minutes	> 65% less time on thermal cycler

*MM- Taq-Polymerase Mastermix SensiMix SYBR No-ROX Kit (Bioline).

### Statistics

Statistical analysis was done using GraphPad PRISM (Version 6.02, GraphPad Software) and Microsoft Excel (Version 2010). Normality of distribution was tested using the Kolmogorov-Smirnov test. Comparison of two groups was done using Mann-Whitney U test for non-normally distributed data, Welch’s t-test or Student’s t-test for normally distributed data with unequal or equal variances, respectively. Bonferroni correction was applied for multiple testing. DNA extraction efficiency was tested by using linear regression as provided in GraphPad PRISM. The ΔCt and ΔΔCt method was calculated as described previously [32].

## Results

**Fig 3:**
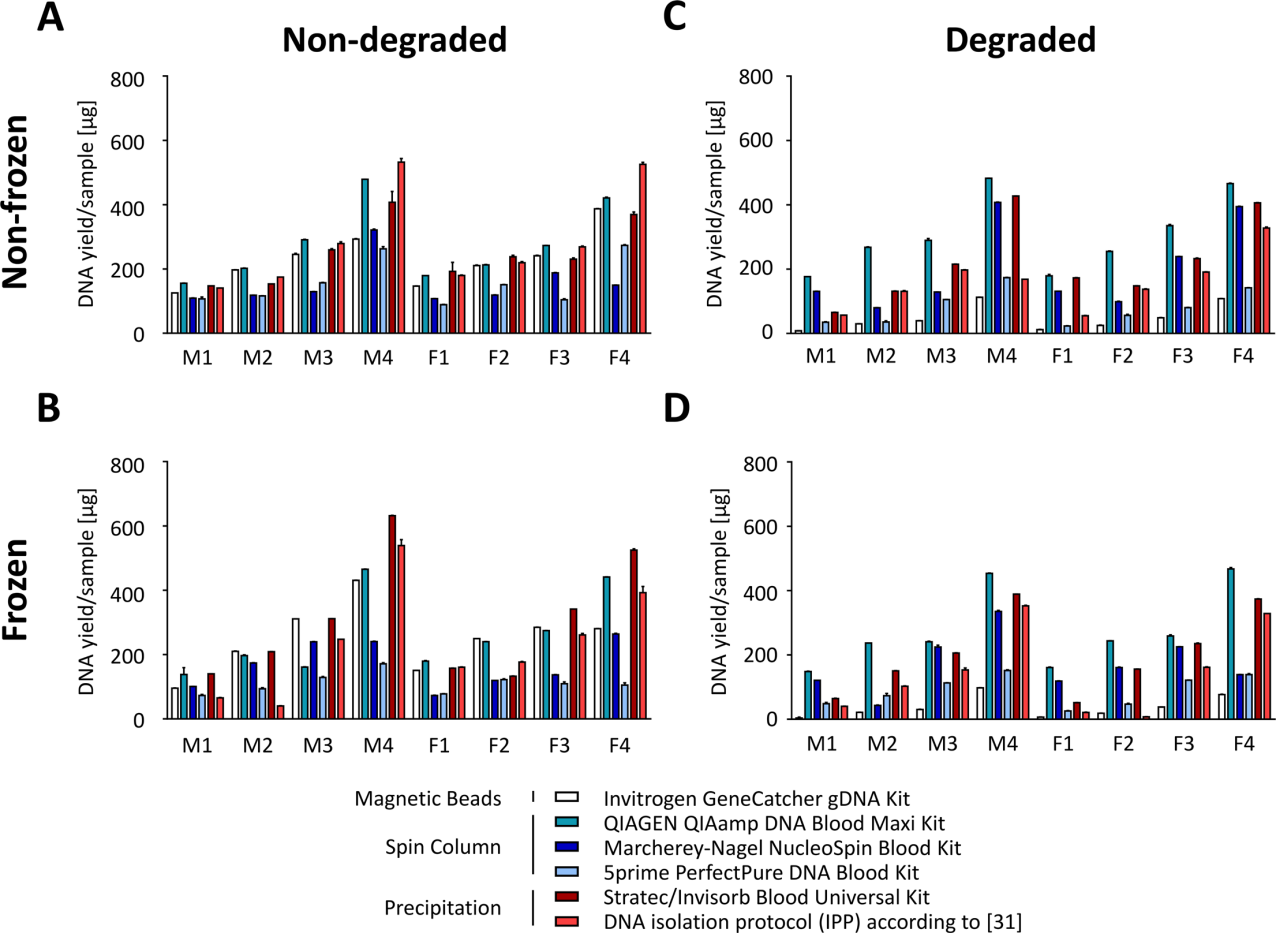
Effect of freezing and degradation on DNA isolation efficiency. Amounts of DNA isolated from 7 ml whole blood aliquots from 8 pools (M/F; n = 4/4) depending on preanalytical conditions. Actinomycin D was used for apoptosis-induced sample degradation. **(A)** Non-frozen, non-degraded samples. **(B)** Frozen, non-degraded samples. **(C)** Non-frozen, degraded samples. **(D)** frozen, degraded samples.

We first assessed the DNA yields obtained with the different isolation protocols. A total of 89–532 μg DNA were isolated with the different DNA isolation kits (Fig 3A). As expected, the isolated DNA amount was proportional to the number of leukocytes in the respective DNA pools. Higher WBC counts, however, decreased the overall DNA extraction efficiency as shown in S2 Fig. Highest DNA amounts were extracted using the IPP (28% higher than average) and the kit from Qiagen (22% higher than average) as well as the kit from Stratec (10% higher than average, Fig 3A). Overall, freezing did not lead to a statistically significant effect on DNA yield, except for the 5prime kit, where a significant 30% decrease in DNA recovery was detected (S2 Fig).

In contrast to freezing, sample degradation significantly impaired DNA recovery (Fig 3C). This effect was particularly pronounced for the Invitrogen kit using magnetic beads (DNA extraction decrease by 79%, p < 10^−4^), but also noticeable when using the 5prime kit (decrease by 48%) and the IPP (decrease by 45%). Freezing in addition to degradation did not further decrease DNA extraction efficiency (overall 9% decrease compared to degraded, non-frozen samples; not significant, Fig 3D, S2 Fig).

**Fig 4:**
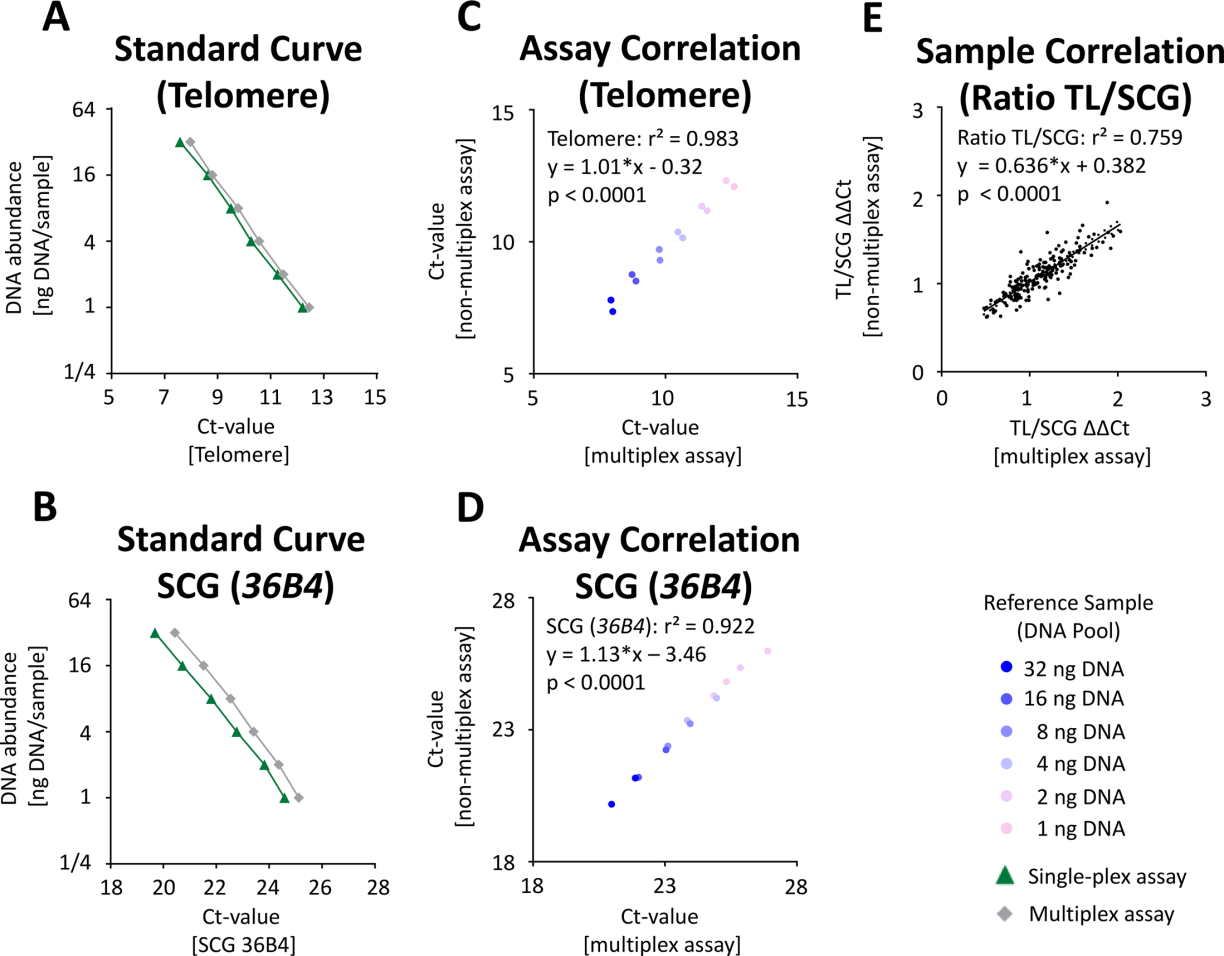
Comparison of TL and SCG quantification using single-plex assays and a multiplex assay. For evaluation of telomeres, qPCRs were performed as described previously [22, 31] with adjustments as described in Material and Methods. Dilutions of a control pool sample were used as standard curve. Standard curves for telomere **(A)** or single copy gene 36B4 **(B)** qPCR assays using the single-plex (green) or multiplex (grey) assay. Correlation between the multiplex and the single-plex assays for TL **(C)** and SCG **(D)**. **(E)** Correlation of TL/SCG ratio in 192 DNA samples.

For TL measurement, an established method [22, 31] was adapted as a multiplex assay as described in the material and methods section. To investigate the technical performance of the multiplex test, we generated a pool from all DNA samples (n = 192) that were previously isolated with the 6 different DNA isolation methods. Using this control pool in different concentrations (1-32ng DNA per qPCR), we showed that the multiplex assay revealed a good linearity when compared to the analysis of TL and SCG *36B4* using single-plex assays (Fig 4A and 4B). qPCR efficiency was comparable between the multiplex and the single-plex assay (> 95%) and a high correlation of TL (r^2^ = 0.983; Fig 4C) and SCG (r^2^ = 0.922; Fig 4D) quantification was shown. Using all 192 DNA samples at a defined concentration (for details see Materials and Methods), a correlation of r^2^ = 0.759 between the TL/SCG ratio of both assays was demonstrated (p < 10^−4^, Fig 4E). Compared to the published assay [31], an 80% reduction of Taq-Polymerase mastermix was achieved when using the multiplex protocol in the 384-well format (Table 2). Furthermore, the input DNA was reduced by > 80% compared to the original assay and the analysis time was reduced by > 60% (Table 2).

**Fig 5:**
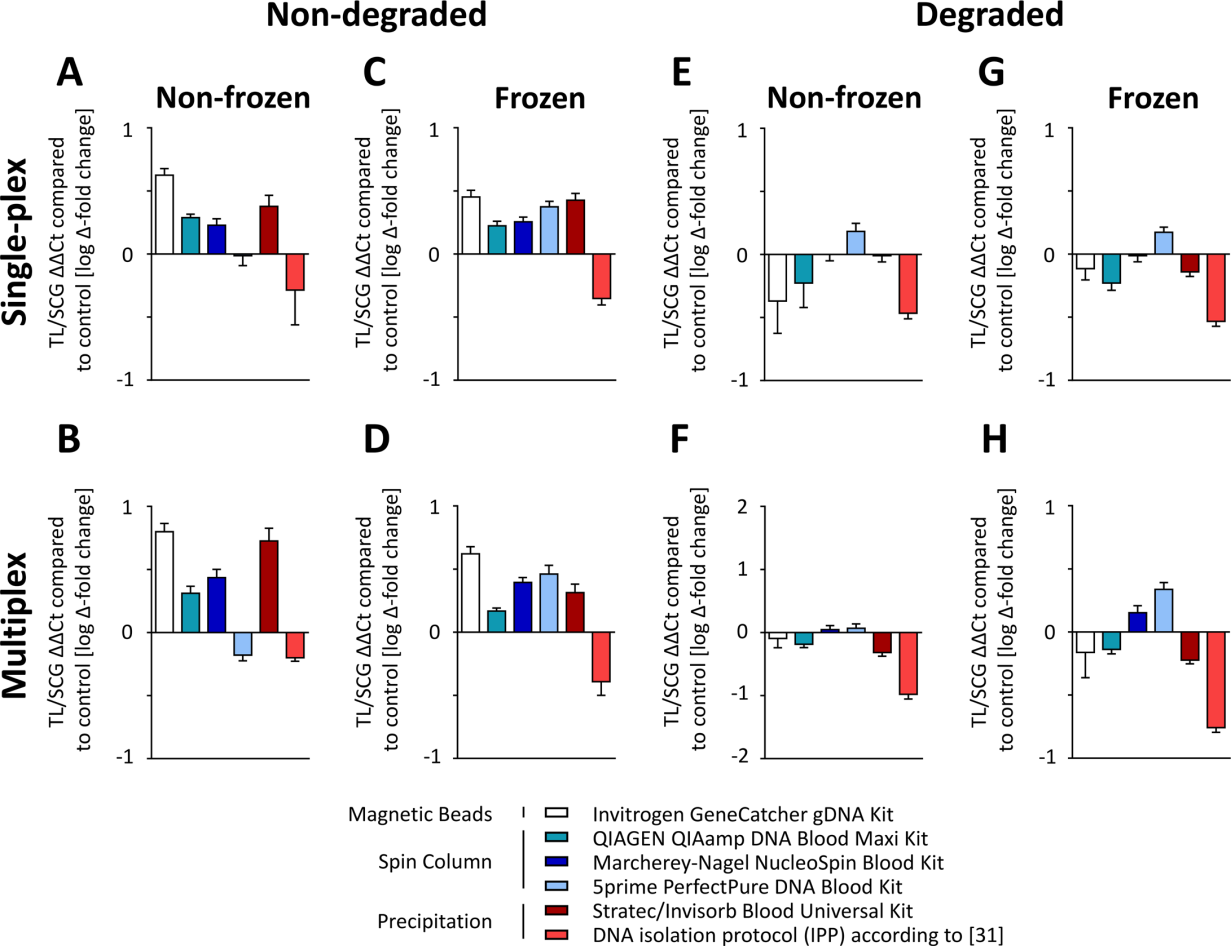
Telomere length is depending of DNA isolation method and preanalytical conditions. 192 samples were analyzed using the single-plex and the multiplex assay. Results from male and female pools (n = 8) per preanalytical condition (n = 4) and DNA isolation (n = 6) method were pooled for analysis of TL/SCG ratio in relation to a pooled reference sample. Absolute differences between both assays were approx. 9% (not significant). **(A,B)** Non-frozen, non-degraded samples. **(C,D)** Frozen, non-degraded samples. **(E,F)** Non-frozen, degraded samples. **(G,H)** frozen, degraded samples.

Next, we investigated the effects of DNA isolation procedures on TL and SCG quantification. To detect whether the single-plex and the multiplex assays performed equally, qPCR analyses were run with both assays (Fig 5). To this end, we analyzed the TL/SCG ratio in 192 DNA samples in relation to a pooled reference sample. As shown in Fig 5A and 5B, different DNA isolation methods significantly affected the TL/SCG ratio already (S1 Table); in non-frozen, non-degraded samples, the strongest changes in TL were ranging from 1.65 to 0.88 when comparing the Invitrogen kit to the IPP protocol. Notably, freezing had generally minor effects on TL quantification (< 5%, not statistically significant; Fig 5C and 5D and S3 Fig). A detailed comparison of TL quantification in frozen and non-frozen samples is provided in S3 Fig. The only kit that revealed significant differences in the TL analysis in frozen and non-frozen samples was the 5prime PerfectPure DNA Blood Kit, which led to a 27% higher telomere abundance in frozen samples. To exclude handling errors as a source of this result, measurements were repeated and revealed comparable results (data not shown).

As shown in Fig 5E–5H, sample degradation had a major impact on TL quantification with an average decrease of 22% compared to non-degraded samples (p < 10^−5^; S4 Fig). This effect was especially pronounced when using the Invitrogen GeneCatcher gDNA Kit, where a lower TL quantification of 40% compared to non-degraded samples was observed. This effect was also strong but less pronounced for the other kits (34% for the Stratec/Invisorb Blood Universal Kit, 25% for the Qiagen QIAamp DNA Blood Maxi Kit, 17% for the IPP and 17% for the Macherey-Nagel NucleoSpin Blood XL Kit compared to non-degraded samples, respectively). The least differences between degraded and non-degraded material was found using the 5prime kit.

## Discussion

In the current study, we systematically evaluated 6 DNA isolation procedures and preanalytical sample treatments such as freezing and apopotosis-induced degradation with respect to their effect on telomere length (TL) and SCG quantification using a high throughput multiplex assay. As major findings, we demonstrate that TL was significantly affected by different DNA isolation methods and that sample degradation reduced TL when using all but one kit. In contrast, sample freezing at -80°C had only minor impacts on TL/SCG quantification with the exception of one DNA isolation method. Our results underscore the importance of standardized preanaytical and analytical procedures when determining TL in clinical and population based studies.

Our study is the largest comparison of DNA isolation methods with respect to TL quantification to date. Since we required 200 ml EDTA-whole blood to compare the effect of four different preanalytical conditions (non-frozen non-degraded, frozen non-degraded, non-frozen degraded and frozen degraded) and six different DNA Maxi isolation kits (7 ml each) on TL quantification, we decided to use pools of anonymized left-over whole blood samples instead of samples from individual probands. A further advantage of pooling samples is that potential bias due to inter-individual biological variance is reduced and pooled samples have also been used by other groups to evaluate technical aspects of TL quantification [23].

With respect to different DNA isolation methods, strongest effects on TL/SCG quantification for non-frozen, non-degraded samples were observed for the kits from Invitrogen (24% higher TL in relation to a pooled reference sample) and the IPP (27% lower TL length than average). In line with results from the current study, a smaller study by Cunningham et al showed significant effects of 3 other different DNA isolation methods on TL [25]. In addition to DNA isolation, preanalytical conditions, such as sample degradation, had major impacts on TL quantification leading to an overall decrease of 22% on average. This effect was especially strong in the Invitrogen GeneCatcher gDNA Kit (40% decrease in relation to a pooled reference sample) and the kit from Stratec (34% decrease in relation to a pooled reference sample). The kit that showed minor differences was from 5prime (4% increase). This kit, however, did not reveal consistent results in non-frozen and frozen samples, where a 27% higher TL was observed after freezing. Koppelstaetter et al have reported changes in TL from tissue after fixation in formaldehyde [33]. Although incubation with the apoptosis-inductor actinomycin D, which was used in the current study, as well as formaldehyde fixation represent extreme, artificial cases of sample degradation, which are probably stronger than expected *in vivo*, it is likely that already slighter sample degradation as it occurs at suboptimal storage conditions [34–36] does affect TL quantification. This is especially important, since the observed biological differences between healthy and diseased patients may vary only in a low percent range [37–41].

In contrast, freezing did not have a significant effect on TL measurement with the exception of the 5prime kit. This is in line with results from Zanet et al., who reported no significant changes in TL between frozen and non-frozen samples. Since this study was only performed using only the QIAcube and QIAamp DNA Mini Kit from Qiagen [42], our study extends this important notion to other DNA isolation kits with the caveat that exceptions might exist.

Especially in large study settings, robust and high throughput methods are warranted for TL analysis. Since qPCR-based methods for TL quantification are widely established because of the analysis speed, scalability and costs-per-sample [2, 43], we have focused on the evaluation of different qPCR methods and have not compared qPCR to Southern blotting or STELA results. We have adapted the method of quantifying telomere length originally published by Cawthon [22, 31] and have established a cost- and time-effective multiplex assay, which can be run in the 384-well format using as little as 10 ng of DNA as opposed to 60 ng [31]. In contrast to a study by Eisenberg et al. [43], we detected no significant changes of TL quantification with respect to the position on the PCR plate speaking in favor of the robustness of the multiplex assay. Additionally, pipetting and other handling errors might also be diminished through the multiplex assay allowing greater standardization and reliability. Since we did not investigate TL using other methods, such as Southern blotting or STELA, it remains to be determined whether results of the current study apply for TL quantification using these techniques, as well.

In the work by Martin-Ruiz et al, the reproducibility of TL quantification in 10 different laboratories was investigated with 3 different techniques (Southern blotting, STELA, qPCR) using 10 human DNA samples and pools [23]. As a main conclusion, the authors suggest to establish a common set of TL standards to improve comparability of TL quantification between methods. Currently, there is an ongoing discussion about the comparability and reproducibility of qPCR, STELA or Southern blotting for TL quantification [23, 44–47]. Whereas the focus of that work was on comparison of different methods for TL quantification, the primary goal of our study was to compare the effects of different DNA isolation protocols and preanalytical conditions. Taken together, previous work and results from our study highlight the need for standardization of the complete process, starting from blood collection, sample storage and DNA isolation, up to TL measurement to obtain reliable TL results, especially in large multi-center study settings.

## Supporting Information

S1 Fig Effect of actinomycin D on white blood cell counts. White blood cell (WBC) counts were measured in samples with and without induction of apoptosis-mediated cell degradation with actinomycin D (ActD; 5 μg/ml) for 24 hours at 37°C. Degradation led to an average 10% WBC decrease. **(A)** The absolute number of leukocytes measured in the sample with or without actinomycin D treatment is shown. **(B)** The relative leukocyte count changes after actinomycin D treatment are presented.
(PDF)

S2 Fig DNA extraction efficiency in dependency of WBC count. The DNA abundance was correlated to the WBC count in each sample. Data are presented as ng DNA per 1000 leukocytes. DNA yields relative to WBC count revealed approx. 3–6 ng DNA/10^3^ leukocytes. Compared to non-frozen, non-degraded samples **(A)**, degradation led to an impaired DNA extraction efficiency in most kits **(C)**, an effect especially pronounced in the Invitrogen GeneCatcher gDNA Kit (DNA extraction efficiency of 21% compared to non-frozen, non-degraded samples). The additional effect of freezing **(B, D)** was minor.
(PDF)

S3 Fig Effects of freezing on TL quantification. When comparing TL of frozen with non-frozen samples, differences were found to be minor (overall approx. 3%) in both the single-plex assay **(A, C)** and the multiplex assay **(B, D)** and irrespective of the analysis of non-degraded **(A, B)** or degraded **(C, D)** samples. The sole exception was the 5prime PerfectPure DNA Blood Kit (27% longer TL in frozen samples). Data are given as fold change of the ratio frozen to non-frozen samples compared to a reference sample.
(PDF)

S4 Fig Effects of degradation on TL quantification. When comparing degraded to non-degraded samples, degradation significantly affected TL measurements (p < 10^−5^) in both the single-plex assay **(A, C)** and the multiplex assay **(B, D)** and irrespective of the analysis of non-frozen **(A, B)** or frozen **(C, D)** samples. These effects were strongest for the Invitrogen GeneCatcher gDNA Kit and the Stratec/Invisorb Blood Universal Kit (40% and 34% decrease, respectively). Data are shown as fold change of the ratio degraded to non-degraded samples compared to a reference sample.
(PDF)

S1 Table Statistically significant differences between TL quantification performed by different DNA extraction kits. Corresponding to Fig 5, levels of significance were calculated for different DNA isolation methods. Bonferroni-correction was applied to correct for multiple testing, thus a p-value of p < 0.00042 was considered to be statistically significant.
(XLSX)
