# Supplementary material for: Preanalytical Conditions and DNA Isolation Methods Affect Telomere Length Quantification in Whole Blood
Source: PLoS One. 2015 Dec 4;10(12):e0143889. doi: 10.1371/journal.pone.0143889 (PMC4670203; doi:10.1371/journal.pone.0143889)
Supplement: S1 Fig — White blood cell (WBC) counts were measured in samples with and without induction of apoptosis-mediated cell degradation with actinomycin D (ActD; 5 μg/ml) for 24 hours at 37°C. Degradation led to an average 10% WBC decrease. (A) The absolute number of leukocytes measured in the sample with or without actinomycin D treatment is shown. (B) The relative leukocyte count changes after actinomycin D treatment are presented. (PDF) [file pone.0143889.s001.pdf]

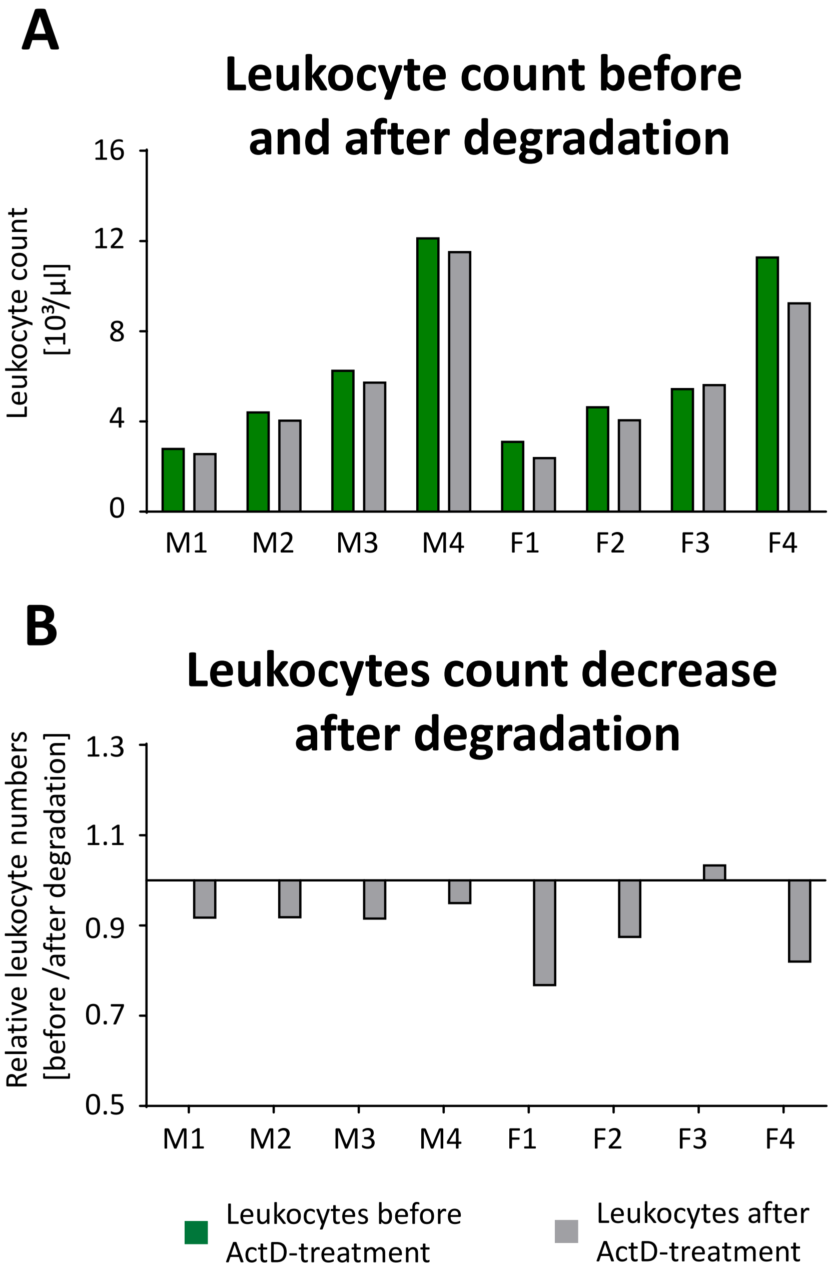

A
Leukocyte count before and after degradation
Leukocyte count [10³/µl]
16
12
8
4
0
M1
M2
M3
M4
F1
F2
F3
F4
B
Leukocytes count decrease after degradation
Relative leukocyte numbers [before /after degradation]
1.3
1.1
0.9
0.7
0.5
M1
M2
M3
M4
F1
F2
F3
F4
Leukocytes before ActD-treatment
Leukocytes after ActD-treatment
