# Supplementary figures and images for: Preanalytical Conditions and DNA Isolation Methods Affect Telomere Length Quantification in Whole Blood
Source: PLoS One. 2015 Dec 4;10(12):e0143889. doi: 10.1371/journal.pone.0143889 (PMC4670203; doi:10.1371/journal.pone.0143889)

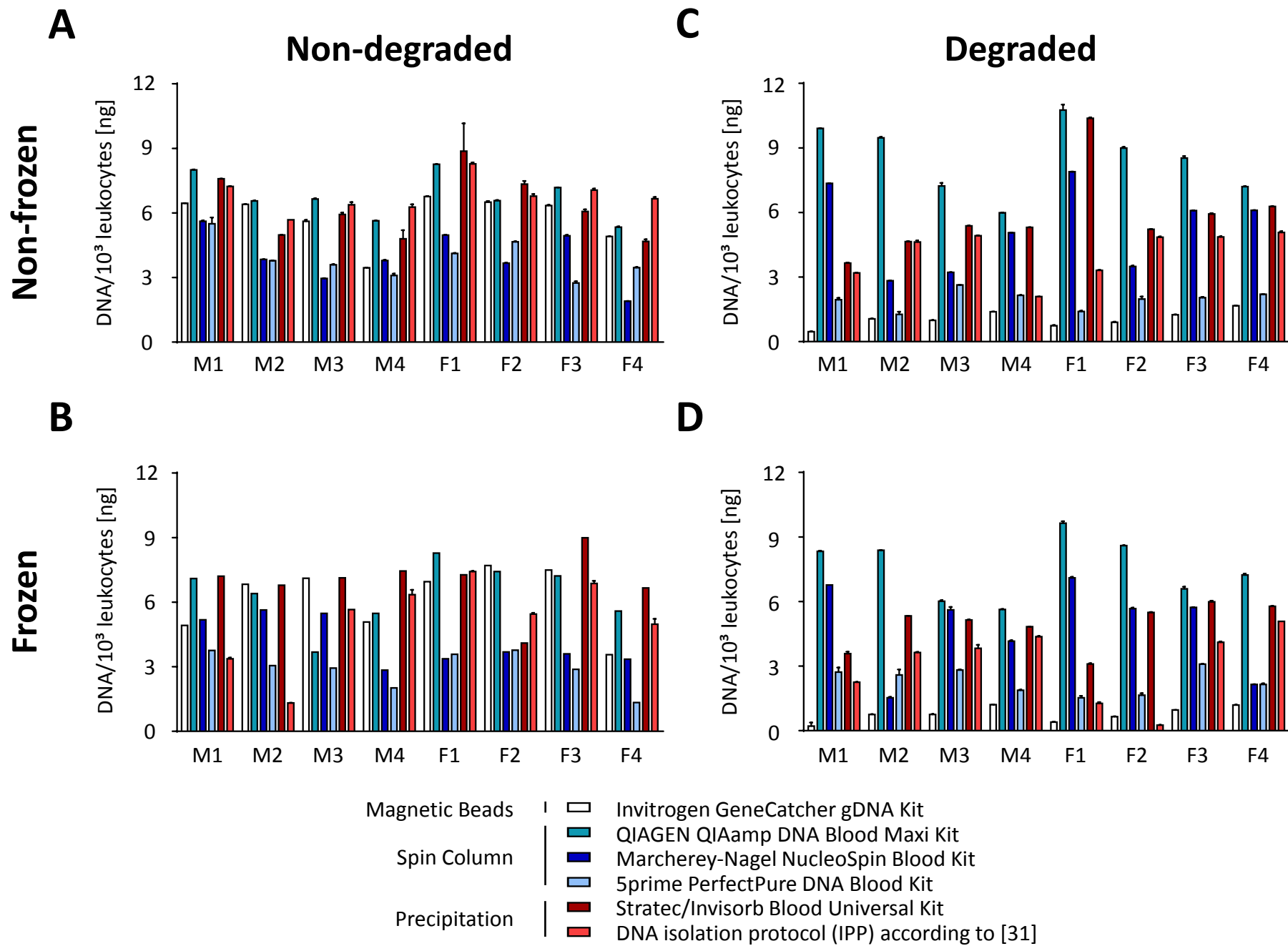

Supplement: S2 Fig — The DNA abundance was correlated to the WBC count in each sample. Data are presented as ng DNA per 1000 leukocytes. DNA yields relative to WBC count revealed approx. 3–6 ng DNA/103 leukocytes. Compared to non-frozen, non-degraded samples (A), degradation led to an impaired DNA extraction efficiency in most kits (C), an effect especially pronounced in the Invitrogen GeneCatcher gDNA Kit (DNA extraction efficiency of 21% compared to non-frozen, non-degraded samples). The additional effect of freezing (B, D) was minor. (PDF) [file pone.0143889.s002.pdf]

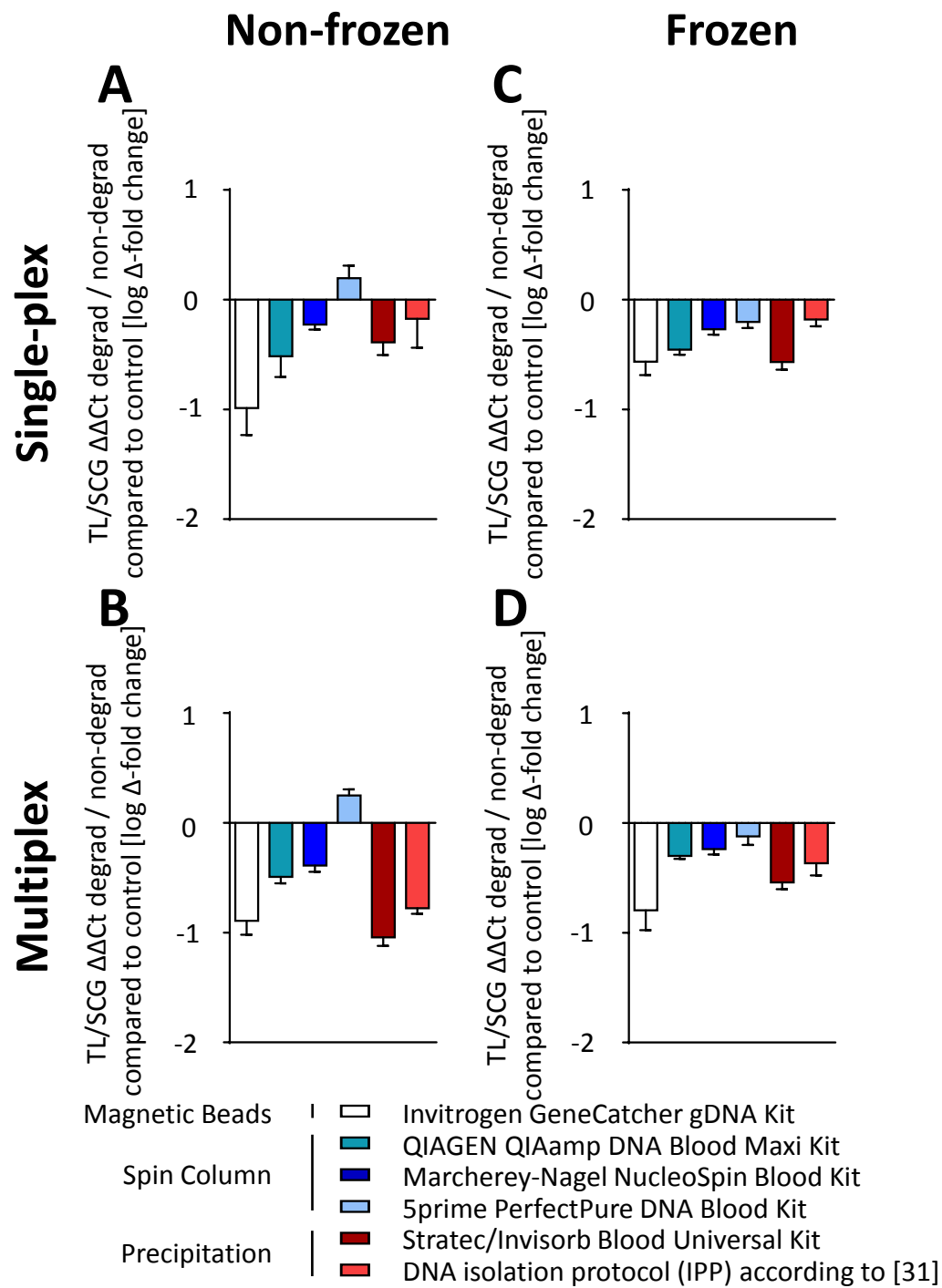

Supplement: S4 Fig — When comparing degraded to non-degraded samples, degradation significantly affected TL measurements (p < 10−5) in both the single-plex assay (A, C) and the multiplex assay (B, D) and irrespective of the analysis of non-frozen (A, B) or frozen (C, D) samples. These effects were strongest for the Invitrogen GeneCatcher gDNA Kit and the Stratec/Invisorb Blood Universal Kit (40% and 34% decrease, respectively). Data are shown as fold change of the ratio degraded to non-degraded samples compared to a reference sample. (PDF) [file pone.0143889.s004.pdf]
