# Supplementary material for: Preanalytical Conditions and DNA Isolation Methods Affect Telomere Length Quantification in Whole Blood
Source: PLoS One. 2015 Dec 4;10(12):e0143889. doi: 10.1371/journal.pone.0143889 (PMC4670203; doi:10.1371/journal.pone.0143889)
Supplement: S3 Fig — When comparing TL of frozen with non-frozen samples, differences were found to be minor (overall approx. 3%) in both the single-plex assay (A, C) and the multiplex assay (B, D) and irrespective of the analysis of non-degraded (A, B) or degraded (C, D) samples. The sole exception was the 5prime PerfectPure DNA Blood Kit (27% longer TL in frozen samples). Data are given as fold change of the ratio frozen to non-frozen samples compared to a reference sample. (PDF) [file pone.0143889.s003.pdf]

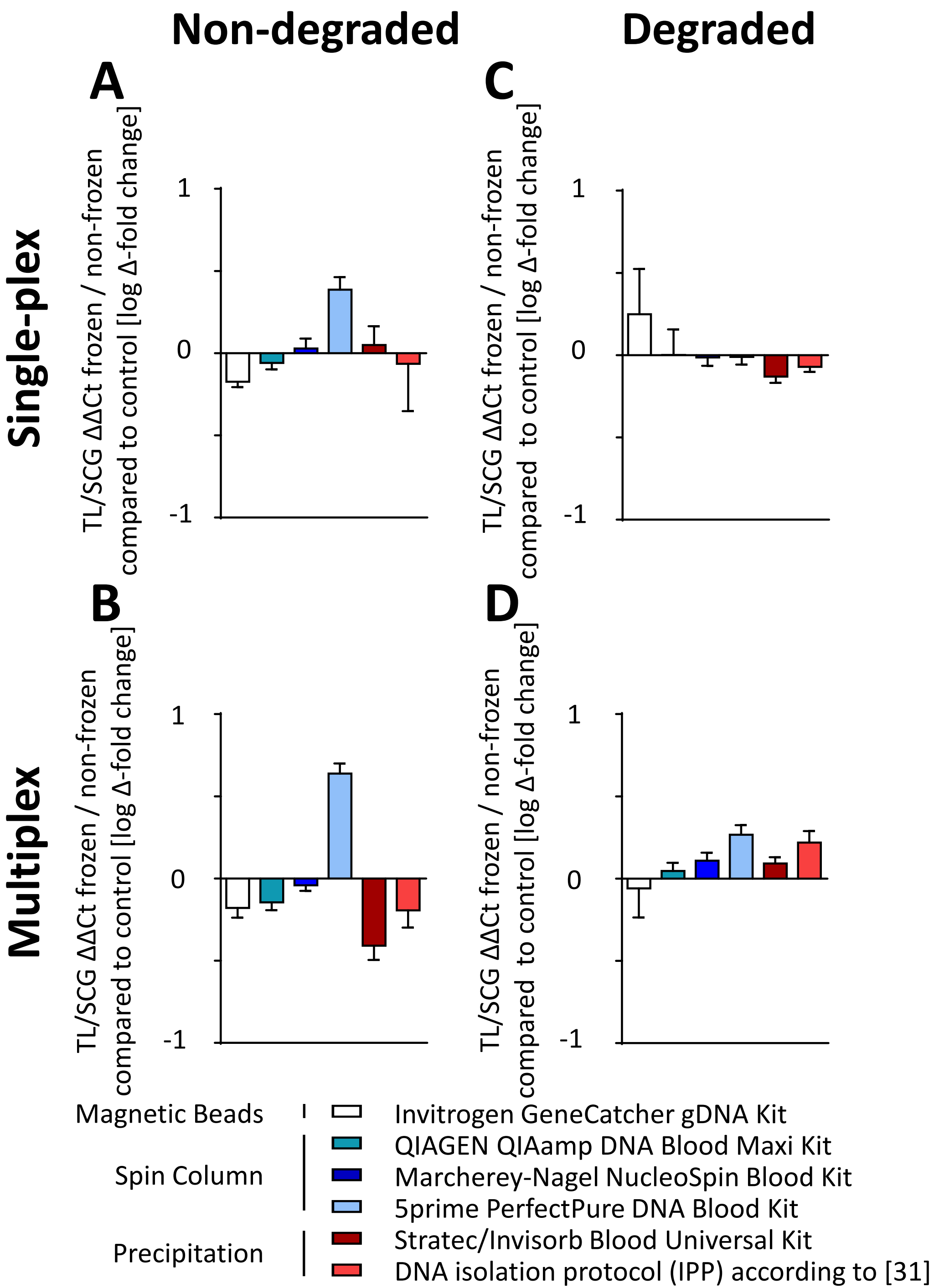

Non-degraded
Degraded
A
C
B
D
Single-plex
Multiplex
TL/SCG ΔΔCt frozen / non-frozen compared to control [log Δ-fold change]
1
0
-1
Magnetic Beads
Spin Column
Precipitation
Invitrogen GeneCatcher gDNA Kit
QIAGEN QIAamp DNA Blood Maxi Kit
Marcherey-Nagel NucleoSpin Blood Kit
5prime PerfectPure DNA Blood Kit
Stratec/Invisorb Blood Universal Kit
DNA isolation protocol (IPP) according to [31]
